# Walking a tightrope: a scoping review of the use, perceptions and experiences of harm reduction strategies in self-harm management

**DOI:** 10.1007/s44192-025-00235-0

**Published:** 2025-08-18

**Authors:** Nina Veetnisha Gunnarsson, Cecilia Moberg

**Affiliations:** 1 Department of Social Work, School of Health and Welfare, Jönköping University, Box 1026, 551 11 Jönköping, Sweden; 2 Department of Health Sciences, The Swedish Red Cross University, Stockholm, Sweden

**Keywords:** Self-harm, Harm reduction, Scoping review, Perceptions, Experiences, Practitioners, Individuals who self-harm

## Abstract

Harm reduction involves safer practices for self-harm, a concept well-established in drug management but less explored in the context of self-harm. This scoping study examines empirical research on harm reduction strategies in self-harm management, focusing on practitioners' and individuals' perspectives. The scoping review follows the Joanna Briggs Institute (JBI) recommendations and analyses internationally peer-reviewed empirical studies. Systematic searches in databases like CINAHL, Scopus, Sociological Abstracts, PsycINFO, and PubMed identified 377 studies, with seven meeting the inclusion criteria. Originating from the UK and focused on the nursing field, recent publications highlighted substitution strategies and alternatives to existing self-harm behaviours, such as snapping an elastic band on the wrist. Both practitioners and those who self-harm used and recommended substitution strategies, but these were also viewed as a form of self-harm. One social scientific study revealed a preference for harm reduction among self-harming individuals, advocating its use to counter oppressive practices. The study metaphorically illustrates practitioners navigating a delicate balance, akin to “walking a tightrope,” striving to prevent harm while recognizing that individuals at times needs to harm themselves in specific situations. The review's emphasis on harm reduction in clinical settings suggests a narrower focus, prompting a call for broader research to understand autonomy and the dynamics of harm reduction in diverse contexts related to self-harm decision-making.

**Supplementary Information:**

The online version contains supplementary material available at 10.1007/s44192-025-00235-0.

## Introduction

Harm reduction strategies, primarily discussed in the context of drug management, aim to minimize the negative impact of harmful behaviours on individuals and society without necessarily reducing the behaviour [1]. [2] defines harm reduction as strategies designed to reduce drug-related harms without reducing drug use.

Drug use, although harmful, does not inherently involve self-harm intent (although it can be used with intent; see for example Connery et al. [3] as is the case with self-harm behaviours such as cutting, burning, or taking deliberate overdoses. Harm reduction strategies for drug use aim to minimize harm without stopping use [4], and can include, for example, providing sterile syringes and allowing drug use in safer environments with medical personnel present [5]. However, such initiatives are often resisted due to fears of condoning harmful behaviour [2].

Harm reduction in self-harm management differs from harm reduction in drug use and can be defined in two main categories: substituting harmful self-harm methods (like cutting or burning) with less harmful alternatives that serve similar purposes, such as self-soothing or emotional regulation, and reducing the harmful effects of self-harm without stopping the behaviour. Substitution strategies can thus be used to reduce self-harm by offering alternative methods that serve similar functions.

Self-harm involves intentional harm to oneself, with or without suicidal intent [6], including direct injuries like cutting or burning and indirect methods like overdosing [7]. Definitions of self-harm vary across regions. In the UK, self-harm is broadly defined to include both direct methods (such as cutting) and indirect methods (such as overdosing) according to NICE [8]. In contrast, Scandinavian countries often use the term Non-Suicidal Self-Injury (NSSI) to refer specifically to deliberate self-inflicted tissue damage without suicidal intent [9]. It is important to note that NSSI is considered a subtype of self-harm, rather than a completely interchangeable term.

Self-harm is often employed as a coping mechanism, helping individuals manage overwhelming emotions and difficult life circumstances [10, 11]. While typically used for short-term relief, it can lead to additional long-term consequences [11]. Although self-harm is not yet an official diagnosis, it has been proposed for inclusion in the DSM-5 [12]. Additionally, self-harm has been described as "contagious," drawing comparisons to the spread of a disease [13].

In contrast with the psychological and medically oriented research social science research suggests viewing self-harm as a social practice rather than an individual pathology [14]. This approach explores broader social and cultural meanings and examines its prevalence across different social groups [15]. Research indicates a shift in perspective, viewing self-harm as an active coping strategy rather than a symptom of illness [16].

Strategies to reduce or stop self-harm have been frequently studied [17]. Fenton and Kingsley [18] found that social support, professional help, and personal coping strategies are crucial for reducing or stopping self-harm. Witt et al. [19] emphasized the effectiveness of cognitive-behavioural therapy (CBT), problem-solving therapy, and various forms of counselling. Nawas et al. [20] highlighted that multifaceted approaches are most effective. However, these studies do not always consider situations in individuals' lives where it is difficult or even impossible to reduce or stop self-harming [21].

What is then harm reduction in connection to self-harm, and what can be learned from the knowledge about harm reduction and self-harm that has been put forth so far? As an extremely understudied area, especially so when it comes to empirical investigations, this scoping review is an attempt to summarise and present what has been done by the academic community to increase the knowledge about what harm reduction in the context of self-harm management means and how it has been targeted. Given that it is such an understudied topic, it becomes even more important to follow up on what has been done, as harm reduction management strategies may have their place in self-harm management, especially if all other attempts to fully reduce or stop self-harm have failed. Changes in self-harm repetition or frequency when considering psychological treatments have also revealed to be small [22, 23].

The aim of this scoping review is to summarise and analyse existing empirical research on the use, perceptions, and experiences of harm reduction strategies in self-harm management, with a focus on identifying key themes and gaps in the literature. The reviewed studies explore how often harm reduction strategies are used or recommended by both practitioners and individuals who self-harm, as well as their perceptions and experiences with these strategies. The scarcity of research on harm reduction in the context of self-harm management underscores a critical gap in the literature that needs to be addressed. Given the growing public health concern surrounding self-harm, examining alternative approaches like harm reduction could offer valuable insights and potentially more effective solutions for some individuals. Self-harm is a complex and often misunderstood practice, one that can provoke anxiety in healthcare professionals [24, 25]. Understanding the views of practitioners and the experiences of individuals who self-harm is essential for delivering person-centred care.

### Aim and research questions

This scoping study summarises and analyses the findings from empirical studies that investigate harm reduction strategies in the management of self-harm. The following research questions was answered:

- How are harm reduction strategies defined in the reviewed studies?
- To what extent are harm reduction strategies used and recommended in the literature, both by practitioners and individuals who self-harm?
- What are the perceptions and experiences of harm reduction strategies, as described by both practitioners and individuals who self-harm?

### Methods

This review aims to describe the findings and range of research that has been undertaken in using harm reduction strategies in managing self-harm. The design of this study employed a scoping review method, utilizing a methodological framework established by Arksey and O'Malley [26] and further refined by Levac et al. [27], along with the methodology outlined for Scoping Reviews by The Joanna Briggs Institute [28]. The overarching goal was to systematically review the available empirical studies concerning harm reduction in the management of self-harm. Scoping reviews are designed to map the existing body of literature, identify key concepts, and highlight gaps in research, rather than to evaluate the quality of individual studies or assess the strength of evidence [26, 28]. The aim of this review was to explore the range of harm reduction strategies in self-harm management, and as such, including studies regardless of their quality allows for a more comprehensive overview of the available literature.

This approach aligns with recommendations from The Joanna Briggs Institute (JBI), which states that scoping reviews are particularly suited for topics where the literature is heterogeneous and emergent, making quality assessment less relevant at this stage of investigation [28]. Therefore, the decision to exclude a formal quality assessment was not due to the limited number of studies available, but because the focus of this scoping review is to provide an initial mapping of the research landscape in this underexplored area.

### Inclusion and exclusion criteria

The criteria for inclusion were based on whether the studies fulfil the following criteria: (a) published in English (or Scandinavian languages), (b) formally use and investigate harm reduction or equivalent terms empirically, e.g., harm minimisation techniques. Some studies may mention but not investigate harm reduction and/or use it without specifying what it entails, which relates to criteria (c) state how harm reduction is defined and (d) having a clear objective of investigating harm reduction strategies to manage self-harm. That is, any studies that were focused on strategies that had the goal of managing self-harm by merely reducing or stopping the use of self-harm were excluded. Finally, to be included in the review, studies must not focus on participants with any learning disabilities or cognitive impairments.

The criteria for exclusion of studies were: (a) languages other than English or Scandinavian, (b) not employing empirical studies to investigate harm reduction, (c) failure to define harm reduction, and (d) absence of an explicit objective to investigate harm-reduction strategies in managing self-harm.

### Search strategies

A variety of electronic databases were searched for the purpose of finding all possible studies carried out on self-harm and harm reduction: PsychINFO, Sociological Abstracts, CINAHL, PubMed, and Scopus, which yielded a total of 377 citations (PsychINFO: 77, Sociological Abstracts: 15, CINAHL: 74, PubMed: 108, and Scopus: 103). The initial screening was conducted by a professional at the university library. A separate search was made in Swedish language (which also captured Danish and Norwegian languages). The search terms employed included combinations of self-injury and harm-reduction, as well as self-harm and harm-minimising techniques, and self-injury and harm-reduction combined with self-harm and minimising techniques. See Table 1 for an example of the search made in CINAHL.

**Table 1:** CINAHL with Full Text (EBSCOhost) n = 74. Date of search: August 10, 2023. Search mode: Boolean/Phrase

	Query	Results
#1	(MH “Injuries, Self-Inflicted”)	3,235
#2	TI ((“self injury” OR “self-injury” OR “self harm”OR self-harm)) AB ((“self injury” OR “self-injury” OR “self harm”OR self-harm))	6,641
#3	#1 OR #2	8,141
#4	(MH “Harm Reduction”)	5,265
#5	TI (“harm reduction” OR “harm minimization”) OR AB (“harm reduction OR “harm minimization”)	4,490
#6	#4 OR #5	7,738
#7	#3 AND #6	74

**Fig. 1:**
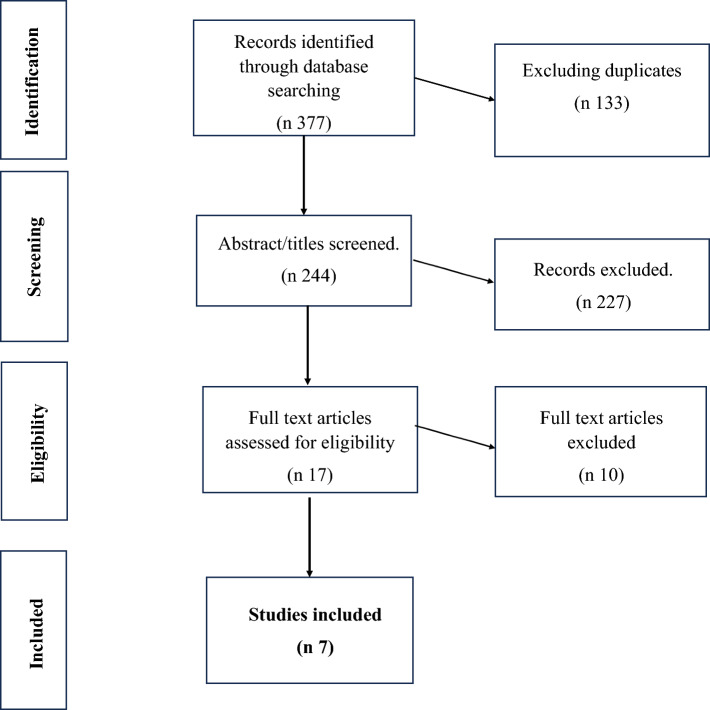
PRISMA flow diagram of paper selection

The titles of the identified studies were then transferred into the Rayyan web application [29] for screening. Subsequently, the two authors independently and blindly assessed the studies based on the abstract. Most studies were excluded (n 227), either because they focused on self-harm per se and not in the context of harm reduction or the studies explored harm reduction in the context of drug treatment (e.g., as non-self-harm activity). Seventeen studies were assessed in full text for eligibility, and ten studies failed to meet the inclusion criteria set for this review (mostly because they were non-empirical studies) (see Fig. 1).

### Data extraction and analysis

Seven studies were eligible for inclusion. All seven studies were thoroughly read and reread several times by the two reviewers. The extracted data, both the quantitative and qualitative data, were analysed independently by the two reviewers and through basic descriptive analysis [28]. The data extracted in this review included: authors, year of publication, which discipline the studies were conducted in, location of studies and self-harm definition used in the studies. We coded the quantitative qualitative and mixed method data independent of each other and discussed the codings afterwards. In the quantitative data the analysis focused upon frequencies of how often one uses or recommend using harm reduction strategies and which strategies one uses or recommend the most. In the qualitative data the focus was on the perceptions and experiences of harm reduction strategies in the management of self-harm, from the viewpoint of both practitioners and individuals who self-harm. These were the findings answering the research question. Any discrepancies or inconsistencies in the analysis of the findings were addressed through communication and clarification of any differing perspectives.

## Results

### Study characteristics

All of the studies on self-harm and harm reduction strategies come from the United Kingdom. Psychiatry, nursing and psychology are the disciplines that foremost write about the issue; however, one social scientific study in sociology/social work were included (see Table 2). The studies were published between 2011 and 2022 but the majority were published between 2020 and 2022. The empirical studies used either a quantitative and qualitative design or a mixed methods design. Definition of self-harm was mainly equivalent to both direct injuries to the body/skin and indirect harm such as self-poisoning, with or without suicidal intents, and is a commonly used definition in the UK. One study, however, used the direct self-injury definition of the deliberate intent to destroy body tissue [30]. The aims range from investigating the characteristics of harm reduction strategies to exploring practitioners’ and self-harming individuals’ views of the approach (see Table 2).

**Table 2:** Scoping review including articles

Study	Location	Discipline	Aims	Setting and sample	Methods	Results
Cliffe et al. [31]	UK	Psychiatry	To determine the proportion of all patients within a large London secondary mental healthcare service who are documented as having used harm-minimisation techniques for self-harm, and to describe the nature of harm-minimisation approaches used and compare the sociodemographic and clinical characteristics of patients who do and do not use harm-minimisation techniques for self-harm	Medical records from Camden and Islington NHS Trust, a secondary mental health service covering 471 000 patients within the boroughs of Camden and Islington 693 of 22,736 patients’ records reporting self-harm (3%)	Mixed methods, quantitative and qualitative Statistical analysis Stata software: descriptive and logistic regression Content analysis of free text entries in electronic records	Self-harm: An intentional act of self-injurywith or without suicidal intent The study found that harm reduction strategies were documented in 3% of self-harming patients' records, with the majority (79%) being female and predominantly younger. These strategies were frequently recommended, with substitution methods being the most common (51.9%) Most patients (92.1%) reported harm reduction strategies as helpful, describing these methods as effective in managing their urge to self-harm. Practitioners' education on harm reduction was also viewed positively
Davies et al. [36]	UK	Psychiatry	To investigate the perceived usefulness of harm reduction techniques for adolescents who self-harm	British early intervention program supporting young people in managing self-harm Eleven service users aged 14–15 years	Qualitative approach: Semi-structured face-to-face interviews Thematic analysis using an inductive approach	Self-harm refers to any non-fatal act performed with the intention of causing harm to oneself The study indicates that harm reduction strategies are recognized and used by adolescents who self-harm, often facilitated by the information and support they receive from practitioners Adolescents viewed harm reduction techniques to manage distress and regain control safely and discreetly, fostering self-compassion and reducing guilt
Haris et al. [35]	UK	Psychiatry	To explore clinicians’ perspectives on the use of harm minimisation techniques for self-harm across a wide range of settings	Primary and secondary care practices in England, Scotland and Wales UK-based clinicians (n = 90; 67% female) working with people who self-harm	Mixed methods approach: Cross-sectional study with purposeful snowball sampling Online survey collecting quantitative data and free-text responses Descriptive statistics and thematic analysis	Self-harm is defined as the intentional act of harming oneself, regardless of motivation and includes self-injury and self-poisoning A significant majority (84%) of the surveyed practitioners reported recommending harm reduction strategies to people who self-harm Practitioners' perceptions of harm reduction strategies were mixed, highlighting both positive and negative outcomes
Hosie and Dickens [30]	UK	Psychiatry	To develop a measure to investigate the attitudes of inpatient mental health service staff and service users towards the management of self-cutting within those settings and the acceptability of management approaches for self-cutting in mental health inpatient settings	Setting: Inpatient mental health settings Sample: Stage One: 27 participants (20 mental health nursing staff and service 7 users) Stage Two: 215 participants (175 mental health practitioners and 40 service users with self-cutting experience)	Two stage mixed-methods design Tool development: literature review and feedback from nurses and users Testing tool: statistical analysis	Self-harm or self-injury “the deliberate destruction of body tissue without conscious intent of suicide” Among mental health nursing staff, 99.1% reported providing therapeutic interventions, while 14.3% had remained present to offer support during self-cutting episodes Among service users, 82.5% had been exposed to passive and active distractions, and 15% had experienced staff presence during cutting Practitioners' and service users' perceptions of harm reduction strategies were generally aligned, with both groups recognizing the effectiveness and acceptability of these interventions
Inckle [33]	UK	Social work/ sociology	To explore a harm-reduction ethos for self-injury and highlight the main themes in a previous project	Participants were accessed through various means, including informal networks, snowballing methods, formal networks, and cold-calling for service-providers and user-led groups with a public profile Participants: Total Participants: 16 individuals Article focus: 6 service-users and 2 service-providers Service Providers: Worked in statutory, private, voluntary, and user-led services Service Users: Individuals with experiences ranging from inpatient psychiatric facilities to user-led peer support groups	Drawing on earlier results from a project using qualitative research with a holistic and harm-reduction approach to self-injury Analysis of the interviews with six service-users and two service-providers	Approaches that accept the need for self-harm at a given time and focus on reducing the risks and damage associated with it The study highlights that traditional prevention and control measures are ineffective and often counterproductive. Harm reduction strategies, although not widely mainstreamed, are recommended and utilized by some service providers and individuals Both groups emphasize the importance of understanding self-harm as a coping mechanism and the need for compassionate, non-judgmental interventions
James et al. [34]	UK	Nursing	To explore nursing practitioners’ perspectives and experiences of harm reduction practices for self-harm in mental health wards	Phase I: Practitioners in 31 acute psychiatric wards in 15 NHS hospitals in the Southeast of England 18 practitioners were selected from the Phase I participants for semi-structured interviews	Mixed methods (quantitative and qualitative) Questionnaire: 395 (63%) STATA version 11 Descriptive analysis Individual semi-structured interviews Thematic analysis	Does not say clearly (Approaches that accept self-harm may be necessary for some individuals and focus on reducing the risks and damage associated with it) The study shows that harm reduction strategies are not widely accepted or implemented, with mixed views among practitioners While some wards had adopted harm reduction practices, others had little to no knowledge of the approach Practitioners expressed concerns that harm reduction could increase the severity and incidence of self-harm, and many viewed it as conflicting with their duty to prevent harm However, those who had implemented harm reduction reported positive outcomes, such as reduced self-harm frequency and improved well-being among service users
Wadman et al. [32]	UK	Psychology	Explore young people’s views of harm minimizing strategies, as a proxy for self-harm	Study 1 (Survey Study): Young people aged 16 to 25 years who reported having ever self-harmed 758 participants were recruited from Child and Adolescent Mental Health Services (CAMHS), Children’s Social Care Services, and the community in the East Midlands, UK Study 2 (Interview study) 45 participants from CAMHS, Children’s Social Care Services, and the community in the East Midlands, UK	Mixed methods Study 1 (Survey Study): 758 participants reporting self-harm selected from a larger study of self-harming behaviour Descriptive statistics Study 2 (Interview study) Semi-structured individual interviews Thematic analysis	Self-harm (any act of self-poisoning or self-injury irrespective of motivation or intent) Harm reduction strategies are defined as approaches that accept self-harm may be necessary for some individuals and focus on reducing the risks and damage associated with it The study indicates mixed use and recommendation of harm reduction strategies among practitioners. While some practitioners and wards have adopted these practices and observed benefits such as reduced self-harm frequency, others remain hesitant or unaware of these strategies. Persons who self-harm also use harm reduction strategies, but with varying perceptions of their effectiveness Practitioners have mixed views on harm reduction, with concerns about increasing self-harm severity and legal responsibilities. many persons who self-harm find harm minimization strategies ineffective or even counterproductive, leading to more severe self-harm

### Types of harm reduction strategies

In two studies harm reduction strategies are categorised in terms of what strategies they are intended for [31, 32] The strategies are used to lessen the harm done to the skin/body by using ways that provide for safer self-harm. The other studies define harm reduction in a single sentence and referenced other study definition, stating, for example, that it involves “practices including providing advice on wound-hygiene, or supplying sterile cutting blades” [30, 33]. Inckle [33, p. 370] emphasizes the goal of harm reduction strategies and that these “accept that someone may need to self-harm at a given point and focus, instead, on supporting that person to reduce the risk and damage inherent in their self-harm.”

It is possible to define harm reduction strategies in mainly two overall categories, whereas the first one relates to substituting the existing self-harm of cutting and burning the skin with less harmful ways that are intended to have a similar functioning as self-soothing, emotional regulation, control, or survival. The second category, on the other hand, aims to minimise the potential harmful effects of self-harm without necessarily promoting a complete cessation of self-harming behaviours. In the context of self-harm, these strategies are closely associated with either changing the actual method of self-harm (substitution) or maintaining the same methods of cutting or burning while consciously striving to reduce harm (e.g., by using clean blades for cutting).The former refers to harm reduction strategies, such as snapping an elastic band onto your wrist, which causes minimal harm but can still induce pain.

Hosie and Dickens [30] describe two substitution methods: sensation proxies and process proxies. These strategies substitute the current form of self-harm by providing a similar sensation (such as pain) or process. For instance, snapping an elastic band against the wrist or using a red pen to draw lines on the body, symbolizing blood. Cliffe et al. [31] refer to this as simulation. According to Wadman et al. [32], harm reduction aims not to replace self-injury methods but to practice them safely, minimizing medical consequences. This includes learning basic body anatomy to avoid severe harm, such as not cutting near major arteries. Harm reduction also involves reducing infection risk by using sterile blades. For Cliffe et al. [31], defer and devoid also involve avoidance strategies, such as removing access to sharp objects, which aim to prevent self-harm rather than reduce harm during the act. Finally, damage limitation focuses on wound care to reduce infection risk and self-manage wounds, with Cliffe et al. [31] also categorizing the use of clean blades under damage limitation.

### Practitioners’ use and perceptions of harm reduction strategies

#### The use of harm reduction strategies and its acceptance

Hosie and Dickens [30] and James et al. [34] recruited mental health nurses who work in inpatient mental health facilities, while Haris et al. [35] recruited any UK-based clinician who had worked with people that self-harm in mental health specialist settings (nurses and GP). These studies focus on the use of practitioner’s harm reduction strategies differently. The studies with the inpatient settings considered harm reduction strategies such as remaining present during a self-cutting episode (14.3% had used being present during a self-cutting episode) [30], providing clean razor blades (6.3% of the nurses had used this strategy), or supporting the clients with the means of self-harming in a safe environment (accepted by 36.3% of the nurses) [34]. Haris et al. [35], who had a more open recruitment, found that 84% of the practitioners recommended substitution approaches to harm reduction, such as using rubber bands and squeezing ice. But very few, on the other hand, had recommended strategies such as clean blades. Providing clean blades and remaining with the patient while cutting were, however, according to Hosie and Dickens [30], ranked low on acceptance compared to other approaches (except for coercive methods such as seclusion of the patient, refusing the patient treatment or psychical restraint). Moreover, many disagreed or strongly disagreed with using such harm reduction strategies or whether they were appropriate for use.

#### Promoting self-harm or developing the therapeutic relationship

James et al.’s [34] interviews with practitioners and Haris et al.’s [35] open-ended questions revealed a similar pattern of why one should or should not use, recommend, or simply accept harm reduction strategies. The practitioners had concerns about the approach regarding two aspects foremost. First, they reflected upon and were worried that harm reduction strategies could result in the self-harm increasing in incidence and severity or even lead to suicide attempts. For example, teaching anatomy was considered inappropriate for individuals who were actively suicidal, as it was thought that they might misuse the information to inflict more harm or attempt suicide. Second, ethical concerns were raised and had to do with the practitioners’ work and their purpose of supporting people and preventing self-harm. Providing their clients with the means to continue hurting themselves in different ways was viewed as promoting or condoning self-harm, which would then go against the notion of preventing harm and supporting a healthy life. However, despite this reluctance, in some mental health wards in James et al.’s [34] study, the practitioners found it impossible to get their clients to stop self-harming and therefore, implemented harm reduction strategies for that reason. These practitioners were also the ones who had less concerns about the approach and had more positive experiences from using such strategies compared to those who had not implemented harm reduction strategies. James et al. [34] found that those practitioners who had used harm reductions strategies had also seen a reduction in incidence and severity of self-harm, and they also stated they had found that it increased the client’s empowerment.

Other reasons for using or recommending harm reduction strategies were their potential to reduce the harm and its positive effects in developing the therapeutic relationship between the practitioners and the clients [35].

### Self-harming individuals use and perceptions of harm reduction strategies

Cliffe et al. [31] investigated the prevalence, as well as the sociodemographic and clinical characteristics of those who self-harm and use harm reduction strategies. They used electronic health records and identified a group of patients who self-harm but do not use harm reduction strategies and used them as a control group (total sample 22,043). They found that only a small number of patients who self-harm used harm reduction strategies (3%) and that they mainly used *substitution strategies* (51.9%, 427/822), which was perceived by the patients as useful in managing their urges to, for example, cut the skin. Nine percent used clean blades and wound care, which refers to limiting the damage done by existing self-harm such as cutting. It should be noted however that 23.5% of those who used harm reduction strategies did not identify what strategy they used. The patients who used harm reduction strategies were more likely to be *younger, female and of white ethnicity,* have more *anxiety related* issues, reported *less hospital admission* for serious self-injury and were le*ss likely to have self- harmed with suicidal intent* than those who did not document the use of harm reduction strategies.

#### Harm reduction as self-harm, safer self-harm or enabling self-care?

Participants in Wadman et al. [32] hardly referred to harm reduction strategies; only 0.9% reported such strategies (of study sample); the majority referred to snapping an elastic band against the skin, but only four (0.5% of study sample) participants did not consider this to be a form of self-harm. The interviewees talked about harm reduction as either ineffective (e.g., snapping an elastic band), effective to a limited extent, or they considered the strategies to be a form of self-harm. The following quote is an example of how one interviewee expressed the ineffectiveness of snapping an elastic band compared to self-harm by, for example, cutting: “…sometimes as much art and pinging elastic bands you can do, there’s nothing quite like the feeling you get from self-harming” (p. 393) [32].

In Wadman et al.’s [32] mixed method study, harm reduction strategies, such as snapping elastic bands against the skin, were viewed as a form of self-harm by the participants (16.2% of 121). Similarly, young people in Davies et al.’s [36] study expressed concerns that harm reduction strategies might encourage more self-harm or drive suicidal individuals to attempt suicide. One interviewee suggested that teaching anatomy “may be a bit problematic if the person is suicidal, because he or she will know exactly where to cut.” Another interviewee thought teaching wound care would encourage self-harm: “If you’re telling people how to clean wound it’s kinda like if someone killed someone, and you’re trying to help them get away with murder…you’re tryna push them towards it…” (p.698). The strategies were also viewed as having short-lived effects [32, 36]. Finally, in the heat of the moment, it was deemed difficult to apply these strategies because of strong self-injurious urges [36].

In accordance with Wadman et al. [32], Davies et al.’s [36] study showed that the young people expressed mixed views on harm reduction strategies and mainly used substitution strategies to manage distress to have a sense of control by self-harming in a safer way. For example, they said they could master one’s self-injury and feel compassionate about themselves in the process, lessening the harm done while having a similar release function as with the self-cutting.

Inckle’s [33] approach to harm reduction strategies is different from the other papers. Coming from a background in sociology and social work, her research on harm reduction demonstrates a clear focus on social science principles and empowering approaches. Participants in her study also had somewhat different backgrounds than participants in other studies, such as activists (peer-led groups), those who had received interventions from formal services (including involuntary hospitalisation), and some with no service contacts at all. The diverse range of participants, including psychiatric survivor activists, contributes to the emphasis on topics such as empowerment. However, Inckle`s [33] also includes a defined theoretical and conceptual framework, enabling her to analyse and interpret research findings from a specific perspective or focal point.

Inckle [33] highlights risk-reducing strategies in self-harm, such as providing anatomical information and clean blades. Participants in the study view self-injury as a response to distress, influenced by external triggers like sexual violence and discrimination, intersecting with class, gender, and sexuality. Inckle emphasizes self-harm as a social phenomenon and advocates for harm reduction strategies as a means of empowering individuals and promoting social justice, shifting focus from mental illness to agency and empowerment.

## Discussion

The reviewed studies highlight substitution strategies as the most common harm reduction methods used or recommended. Although both practitioners and those who self-harm acknowledge other approaches aimed at minimizing potential harmful effects, these are very rarely used. For example, providing clean razor blades and allowing self-harm in a secure setting with medical personnel present are seldom recommended in self-harm management. Substitution strategies are employed and recommended more frequently, but they are still not often routinely applied in either outpatient or inpatient care. This may be because both practitioners and individuals who self-harm sometimes perceive all harm reduction strategies as another form of self-harm.

In general, there is a hesitancy among both practitioners and those engaging in self-harm if it is beneficial and “right” to use harm reduction strategies in self-harm management. At the same time, there are practitioners who demonstrate a willingness to endorse harm reduction strategies, particularly in situations where all other attempts to reduce harm through cessation of self-injury have proven ineffective. Most studies nevertheless reveal that both groups are worried about using harm reduction strategies. Sullivan [37] argues that health practitioners’ reluctance to use harm reduction strategies is embedded in the context of them being “increasingly risk conscious and more aware of legal implications of their work” (p. 9), because as he writes practitioners are afraid of being accused or blamed for doing something that will risk serious harm or even their patient’s life.

As there is no research evidence that these strategies work to reduce incidents of self-harm and self-harm severity and frequency, it is up to the individual healthcare professional or team of professionals to decide whether there is enough clinical evidence for the use of these strategies and what value these strategies have for their patients [38]. There is on the whole little evidence that current interventions have effects on the cessation of self-harm [23]. Psychological treatment has some effects on the repetition and frequency of self-harm, but they are small [22, 39].

Researchers have debated the use of harm reduction strategies and Sullivan [40, 41] considers the ethical justification of allowing people to self-harm in a safe environment and asserts that health professionals sometimes should let them do so. He concurs that preventing self-harm fails to respect the individual’s autonomy and conveys the message that the individual’s own choice is not valuable. Thus, harm reduction strategies, in this respect, can function to improve the therapeutic relationship. Pickard and Pearce [42], on the other hand, allude that facilitating the therapeutic relationship by supporting individuals to self-harm is just another way of sanctioning it and that it may give the patient the message that you are not worth saving. They are particularly concerned with using harm reduction strategies in inpatient settings.

The debate between researchers as presented above and the perceptions of practitioners and those who self-harm suggests that harm reduction strategies in the management of self-harm are highly controversial. They are used by both practitioners and those who self-harm, but not to a great extent. Nonetheless, there is a great ambivalence in whether it is considered appropriate and ethically “right” to apply them to self-harm. However, in most studies, self-harm is understood within a psychomedical paradigm, in which the ethical guideline is to do no harm. It is logical then that allowing people to self-harm, even in a safe way, would stir up emotional and ethical concerns. But self-harm also relates to oppressional structures of gender, class, and sexuality [43]. This indicates that several individuals who self-harm are already exposed to discriminatory practices, and harm reduction within this context means a way for these individuals to reclaim power and agency over their lives and their bodies.

All studies reviewed come from UK which makes it possible to relate to the debate and certain advocacy of self-harm reduction strategies in the UK among, for example, practitioners, individuals who self-harm, support groups, and researchers. For example, the National Institute for Health and care Excellence [6] has provided clinical guidelines that make recommendations for practitioners to use harm reduction strategies, at least for cases where self-harm is likely to persist and be done repeatedly. Harm reduction strategies for self-harm are becoming more common in the UK due to sociocultural factors like the recognition that traditional methods focused on stopping self-harm entirely may not work for everyone and could increase distress. Instead, harm reduction aims to minimize harm while addressing emotional regulation issues. The UK's mental health services, particularly in urban areas like London, support this approach, with a growing emphasis on patient autonomy and personalized care models promoting its adoption.

Additionally, the strong presence and influence of the psychiatric survival movement in the UK may have contributed to the focus on these strategies. The psychiatric survivor's movement, also known as the consumer/survivor/ex-patient movement, is a diverse association of individuals who either currently use mental health services (referred to as consumers or service users), consider themselves survivors of psychiatric interventions, or identify as ex-patients of mental health services. Still, even in the UK, where they practice harm reduction in both outpatient and inpatient treatment, the usefulness and appropriateness of these strategies are far from clear or straightforward.

In terms of drug harm reduction, syringe exchange programs do effectively prevent certain forms of harm, such as the transmission of blood-related diseases [44], even though they do not address the actual method of drug use. The equivalent of syringe programs in self-harm management would be providing clean blades. However, it is important to note that the act of cutting and burning oneself is inherently harmful, unlike providing clean syringes to drug users, which is a harm reduction strategy aimed at preventing future harm. Indeed, there is a fundamental difference between intentional self-harm activities, where the individual aims to cause harm to themselves, and unintentional harm caused by drug use. In self-harm, the intention is to inflict harm as a coping mechanism or expression of emotional distress [10]. On the other hand, harm reduction strategies in drug use focus on minimising unintended harm and promoting safer practices. The distinction lies in the intentional nature of self-harm versus the unintentional harm associated with drug use.

This review indicates that practitioners find harm reduction strategies clinically useful and that these strategies have value for those who engage in harm reduction activities. However, it also shows, perhaps more clearly, that there are ethical dilemmas, individual perceptions, and judgments among both professionals and those with experience of self-harm, as well as real concerns about the consequences of'allowing' people to continue hurting themselves. This means that it is primarily the experiences people have regarding the usefulness of harm reduction strategies, and the perceptions that practitioners and individuals who self-harm hold about whether harm reduction is an ethically defensible management strategy, that direct the actual use and experiences of harm reduction in self-harm management. Best practice should be based on research evidence, clinical expertise, and the value the practice has for the patient.

Harm reduction strategies may enable people who self-harm to take control of their own lives and bodies, and work to reduce their need for self-harm. But if it does not, can healthcare and other professionals encountering self-harming individuals, help in supporting a less medically dangerous and severe way of hurting themselves? For example, with direct self-harm, such as cutting one's skin, anatomical knowledge about the body may offer a way to reduce the harm of cutting.

Considering what has been elucidated in this review, one can metaphorically state that practitioners need to “walk a tightrope” between preventing and doing no harm and of perhaps sometimes accepting and allowing people to do harm to themselves. There is also tension between medical studies of harm reduction, where these strategies are often set in a clinical setting and from a patient perspective, and the more socially oriented study. However, only one study embeds harm reduction strategies in the context of individual agency and empowerment.

### Study limitations

This scoping review is based on a small number of empirical studies about self-harm and harm-reduction, as there is to our knowledge no more empirically guided studies on the subject. There is thus a limitation to what possible conclusion that can be drawn from this scoping review. What this scoping review can shed light on is to what extent different practitioners or individuals who self-harm actually use or recommend harm reduction strategies, and especially what their perceptions and experiences are of using such strategies in managing self-harm.

The lack of empirical studies in this area may be due to various factors, including limited research interest, ethical considerations, or challenges in conducting such studies. It highlights the need for future research to investigate and evaluate the effectiveness of harm reduction approaches in addressing self-harm behaviours. Despite some progress in the field, there remains a limited knowledge base regarding how practitioners and individuals who self-harm engages with, perceive, and experience these management strategies. By gaining insights from both practitioners and individuals with lived experiences, the understanding of the practical implementation and perceived effectiveness of these strategies can be enhanced, along with their impact on the well-being of those who self-harm. Such knowledge can inform the development of more tailored and person-centred approaches to self-harm management, ultimately improving support and care for individuals who self-harm.

## Conclusion

This scoping review aims to organize existing knowledge, identify areas requiring further research, and support clinicians in developing compassionate and practical strategies for managing self-harm.

The reviewed studies primarily highlight substitution strategies as harm reduction, even though they acknowledge other strategies aiming to minimize potential harmful effects. Even though substitution strategies are utilized to some extent, they are often not routinely applied in either open care facilities or inpatient care. This may be because they are perceived by both practitioners and individuals who self-harm as another form of self-harm.

The study underscores the limited use of harm reduction strategies in self-harm management, with substitution strategies being the most applied, though even these are not routinely implemented in clinical settings. More controversial strategies, such as supervised self-harm with clean instruments, are rarely utilized due to ethical concerns and the perception that they perpetuate self-harm behaviour. This reluctance to adopt a broader harm reduction approach highlights the tension between ensuring patient safety and respecting individual coping mechanisms. The review’s findings indicate a significant gap in research on harm reduction strategies, calling for more in-depth exploration to assess the effectiveness, ethical considerations, and potential for integrating these approaches into standard care. Addressing this gap could lead to more comprehensive and compassionate care strategies that meet the diverse needs of individuals who self-harm.

To further research in this area, a Participatory Action Research (PAR) framework [45] which involves participants in the research process, could be useful. PAR allows participants to co-design the study and provide ongoing feedback. This approach is particularly relevant for harm reduction, where individuals who self-harm may have strong views on their own care. PAR has the potential to empower participants and can lead to more applicable and ethical interventions by incorporating their lived experiences and preferences. It also allows for the direct exploration of changes in self-agency and personal empowerment.

Ultimately, this scoping review highlights a significant gap in the existing literature on harm reduction in self-harm management (Supplementary Material 1), underscoring the urgent need for comprehensive, empirical research to better understand and evaluate the effectiveness of these strategies in diverse clinical and social contexts.

## Supplementary Information

Below is the link to the electronic supplementary material.

Supplementary Material 1

## Data Availability

No datasets were generated or analysed during the current study.

## References

[1] Roe G. Harm reduction as paradigm: Is better than bad good enough? The origins of harm reduction. Crit Public Health. 2005;15(3):243–50.

[2] Weatherburn D. Dilemmas in harm minimization. Addiction. 2009;104(3):335–9. 18855811 10.1111/j.1360-0443.2008.02336.x

[3] Connery HS, Taghian N, Kim J, Griffin M, Rockett IRH, Weiss RD, et al. Suicidal motivations reported by opioid overdose survivors: a cross-sectional study of adults with opioid use disorder. Drug Alcohol Depend. 2019;205:107612. 31627077 10.1016/j.drugalcdep.2019.107612 PMC6929689

[4] Marlatt GA, Witkiewitz K. Update on harm-reduction policy and intervention research. Annu Rev Clin Psychol. 2010;6(1):591–606. 20192791 10.1146/annurev.clinpsy.121208.131438

[5] Shorter GW, McKenna-Plumley PE, Campell KB, Keemink JR, Scher BD, Cutter S, et al. Overdose prevention centres, safe consumption sites, and drug consumption rooms: a rapid evidence review. London: University of West London; 2023.

[6] Evidence review for harm minimisation strategies: Self-harm: assessment, management and preventing recurrence: Evidence review L. London: National Institute for Health and Care Excellence (NICE); 2022. (NICE Evidence Reviews Collection). http://www.ncbi.nlm.nih.gov/books/NBK588192/36595594

[7] Claassen CA, Trivedi MH, Shimizu I, Stewart S, Larkin GL, Litovitz T. Epidemiology of nonfatal deliberate self-harm in the united states as described in three medical databases. Suicide Life Threat Behav. 2006;36(2):192–212. 16704324 10.1521/suli.2006.36.2.192

[8] National Collaborating Centre for Mental Health (UK). Self-Harm: Longer-Term Management. Leicester (UK): British Psychological Society (UK); 2012. (National Institute for Health and Care Excellence: Guidelines). http://www.ncbi.nlm.nih.gov/books/NBK126777/23534084

[9] Mohl B, La Cour P, Skandsen A. Non-suicidal self-injury and indirect self-harm among Danish high school students. Scand J Child Adolesc Psychiatry Psychol. 2013;2(1):11–8.

[10] Adler PA, Adler P. The tender cut: inside the hidden world of self-injury. New York: New York University Press; 2011.

[11] Taylor PJ, Jomar K, Dhingra K, Forrester R, Shahmalak U, Dickson JM. A meta-analysis of the prevalence of different functions of non-suicidal self-injury. J Affect Disord. 2018;227:759–69. 29689691 10.1016/j.jad.2017.11.073

[12] American Psychiatric Association. (eds). Diagnostic and statistical manual of mental disorders: DSM-5-TR. Fifth edition, Text revision. Washington DC: American Psychiatric Association Publishing; 2022.

[13] Hasking P, Boyes M. Cutting words: a commentary on language and stigma in the context of nonsuicidal self-injury. J Nerv Ment Dis. 2018;206(11):829–33. 30371637 10.1097/NMD.0000000000000899

[14] Brossard B, Steggals P. The sociological implications of taking self-injury as a practice: an author meets critic interview. Soc Theory Health. 2020;18(3):211–23.

[15] Chandler A, Myers F, Platt S. The construction of self-injury in the clinical literature: a sociological exploration. Suicide Life Threat Behav. 2011;41(1):98–109. 21309828 10.1111/j.1943-278X.2010.00003.x

[16] Woodley S, Hodge S, Jones K, Holding A. How individuals who self-harm manage their own risk—‘I Cope Because I Self-Harm, and I Can Cope with my Self-Harm.’ Psychol Rep. 2021;124(5):1998–2017. 32718228 10.1177/0033294120945178 PMC8422773

[17] Brennan CA, Crosby H, Sass C, Farley KL, Bryant LD, Rodriquez-Lopez R, et al. What helps people to reduce or stop self-harm? A systematic review and meta-synthesis of first-hand accounts. J Public Health. 2023;45(1):154–61. 10.1093/pubmed/fdac022 PMC10017083 35211734

[18] Fenton C, Kingsley E. Scoping review: alternatives to self-harm recommended on mental health self-help websites. Int J Ment Health Nurs. 2023;32(1):76–94. 36104975 10.1111/inm.13067

[19] Hawton K, Witt KG, Salisbury TLT, Arensman E, Gunnell D, Hazell P, et al. Psychosocial interventions following self-harm in adults: a systematic review and meta-analysis. Lancet Psychiatry. 2016;3(8):740–50. 27422028 10.1016/S2215-0366(16)30070-0

[20] Nawaz RF, Anderson JK, Colville L, Fraser-Andrews C, Ford TJ. Review: interventions to prevent or manage self-harm among students in educational settings—a systematic review. Child Adolesc Ment Health. 2024;29(1):56–69. 36625166 10.1111/camh.12634

[21] Peel-Wainwright K, Hartley S, Boland A, Rocca E, Langer S, Taylor PJ. The interpersonal processes of non-suicidal self-injury: a systematic review and meta-synthesis. Psychol Psychother Theory Res Pract. 2021;94(4):1059–82. 10.1111/papt.12352 34090311

[22] Fox KR, Huang X, Guzmán EM, Funsch KM, Cha CB, Ribeiro JD, et al. Interventions for suicide and self-injury: a meta-analysis of randomized controlled trials across nearly 50 years of research. Psychol Bull. 2020;146(12):1117–45. 33119344 10.1037/bul0000305

[23] Saunders KE, Smith KA. Interventions to prevent self-harm: What does the evidence say? Evid Based Ment Health. 2016;19(3):69–72. 27436413 10.1136/eb-2016-102420 PMC10699448

[24] Hadfield J, Brown D, Pembroke L, Hayward M. Analysis of accident and emergency doctors’ responses to treating people who self-harm. Qual Health Res. 2009;19(6):755–65. 19429768 10.1177/1049732309334473

[25] Inckle K. At the cutting edge: creative and holistic responses to self-injury. Creat Nurs. 2010;16(4):160–5. 21140868 10.1891/1078-4535.16.4.160

[26] Arksey H, O’Malley L. Scoping studies: towards a methodological framework. Int J Soc Res Methodol. 2005;8(1):19–32.

[27] Levac D, Colquhoun H, O’Brien KK. Scoping studies: advancing the methodology. Implement Sci. 2010;5(1):69. 20854677 10.1186/1748-5908-5-69 PMC2954944

[28] Peters MDJ, Marnie C, Tricco AC, Pollock D, Munn Z, Alexander L, et al. Updated methodological guidance for the conduct of scoping reviews. JBI Evid Synth. 2020;18(10):2119–26. 33038124 10.11124/JBIES-20-00167

[29] Ouzzani M, Hammady H, Fedorowicz Z, Elmagarmid A. Rayyan—a web and mobile app for systematic reviews. Syst Rev. 2016;5(1):210. 27919275 10.1186/s13643-016-0384-4 PMC5139140

[30] Hosie L, Dickens GL. Harm-reduction approaches for self-cutting in inpatient mental health settings: development and preliminary validation of the Attitudes to Self-cutting Management (ASc-Me) Scale. J Psychiatr Ment Health Nurs. 2018;25(9–10):531–45. 30256488 10.1111/jpm.12498

[31] Cliffe C, Pitman A, Sedgwick R, Pritchard M, Dutta R, Rowe S. Harm minimisation for the management of self-harm: a mixed-methods analysis of electronic health records in secondary mental healthcare. BJPsych Open. 2021;7(4): e116. 34172102 10.1192/bjo.2021.946 PMC8269923

[32] Wadman R, Nielsen E, O’Raw L, Brown K, Williams AJ, Sayal K, et al. *These Things Don’t Work*.” Young people’s views on harm minimization strategies as a proxy for self-harm: a mixed methods approach. Arch Suicide Res. 2020;24(3):384–401. 31322056 10.1080/13811118.2019.1624669

[33] Inckle K. The first cut is the deepest: a harm-reduction approach to self-injury. Soc Work Ment Health. 2011;9(5):364–78.

[34] James K, Samuels I, Moran P, Stewart D. Harm reduction as a strategy for supporting people who self-harm on mental health wards: the views and experiences of practitioners. J Affect Disord. 2017;214:67–73. 28284098 10.1016/j.jad.2017.03.002

[35] Haris AM, Pitman A, Mughal F, Bakanaite E, Morant N, Rowe SL. Harm minimisation for self-harm: a cross-sectional survey of British clinicians’ perspectives and practices. BMJ Open. 2022;12(6): e056199. 35980724 10.1136/bmjopen-2021-056199 PMC9171231

[36] Davies J, Pitman A, Bamber V, Billings J, Rowe S. Young peoples’ perspectives on the role of harm reduction techniques in the management of their self-harm: a qualitative study. Arch Suicide Res. 2022;26(2):692–706. 33027597 10.1080/13811118.2020.1823916

[37] Sullivan PJ. Risk and responding to self injury: is harm minimisation a step too far? J Ment Health Train Educ Pract. 2019;14(1):1–11.

[38] Titler MG. The Evidence for Evidence-Based Practice Implementation. I: Hughes RG. (eds). Patient safety and quality: an evidence-based handbook for nurses. Rockville (MD): Agency for Healthcare Research and Quality (US); 2008. (Advances in Patient Safety). http://www.ncbi.nlm.nih.gov/books/NBK2659/

[39] Witt KG, Hetrick SE, Rajaram G, Hazell P, Taylor Salisbury TL et al. Townsend E, Psychosocial interventions for self-harm in adults. Cochrane Common Mental Disorders Group, redaktör. Cochrane Database Syst Rev. 2021. 10.1002/14651858.CD013668.pub2 10.1002/14651858.CD013668.pub2 PMC8094743 33884617

[40] Sullivan PJ. Should healthcare professionals sometimes allow harm? The case of self-injury. J Med Ethics. 2017;43(5):319–23. 28183785 10.1136/medethics-2015-103146

[41] Sullivan PJ. Sometimes, not always, not never: a response to Pickard and Pearce. J Med Ethics. 2018;44(3):209–10. 28912288 10.1136/medethics-2017-104343

[42] Pickard H, Pearce S. Balancing costs and benefits: a clinical perspective does not support a harm minimisation approach for self-injury outside of community settings. J Med Ethics. 2017;43(5):324–6. 28183784 10.1136/medethics-2017-104152

[43] Inckle K. Inequality, distress and harm-reduction: a social justice approach to self-injury. Soc Theory Health. 2020;18(3):224–39.

[44] Yeh PT, Yang X, Kennedy CE, Armstrong KA, Fonner VA, Sherryn, et al. The impact of needle and syringe exchange programs on HIV-related risk behaviors in low- and middle-income countries: a systematic review and meta-analysis examining individual-versus community-level effects. AIDS Behav. 2023;27(10):3306–31. 37046029 10.1007/s10461-023-04051-x PMC10524190

[45] Hensler L, Frenk A, Merçon J. Participatory action research. I: and book Transdisciplinary Learning. Bielefeld: Transcript Verlag, 2023.

