# Supplementary material for: Walking a tightrope: a scoping review of the use, perceptions and experiences of harm reduction strategies in self-harm management
Source: Discov Ment Health. 2025 Aug 18;5(1):125. doi: 10.1007/s44192-025-00235-0 (PMC12361002; doi:10.1007/s44192-025-00235-0)
Supplement: Supplementary file 1 — Supplementary Material 1 [file 44192_2025_235_MOESM1_ESM.docx]

**Search strategies supplementary file**

**CINAHL with Full Text (EBSCOhost) n=74**Date of search: August 10, 2023
Search mode: Boolean/Phrase

|  | **Query** | **Results** |
| --- | --- | --- |
| #1 | (MH "Injuries, Self-Inflicted") | 3,235 |
| #2 | TI ( (“self injury” OR self-injury OR “self harm” OR self-harm) ) OR AB ( (“self injury” OR self-injury OR “self harm” OR self-harm) ) | 6,641 |
| #3 | #1 OR #2 | 8,141 |
| #4 | (MH "Harm Reduction") | 5,265 |
| #5 | TI ( ”harm reduction” OR ”harm minimisation” ) OR AB ( ”harm reduction” OR ”harm minimisation” ) | 4,490 |
| #6 | #4 OR #5 | 7,738 |
| #7 | #3 AND #6 | 74 |

**PsycInfo (ProQuest) n=77**Date of search: August 10, 2023

|  | **Query** | **Results** |
| --- | --- | --- |
| #1 | MAINSUBJECT.EXACT.EXPLODE("Nonsuicidal Self-Injury") | 7,860 |
| #2 | tiab((“self injury” OR self-injury OR “self harm” OR self-harm) ) | 10,294 |
| #3 | #1 OR #2 | 13,227 |
| #4 | MAINSUBJECT.EXACT.EXPLODE("Harm Reduction") | 4,708 |
| #5 | tiab(”harm reduction” OR ”harm minimisation”) | 4,909 |
| #6 | #4 OR #5 | 7,160 |
| #7 | #3 AND #6 | 77 |

**PubMed (National Library of Medicine) n=108**Date of search: August 10, 2023

|  | **Query** | **Results** |
| --- | --- | --- |
| #1 | "Self-Injurious Behavior"[Mesh] | 83,799 |
| #2 | ("self injury"[Title/Abstract] OR self-injury[Title/Abstract] OR "self harm"[Title/Abstract] OR self-harm[Title/Abstract]) | 11,942 |
| #3 | #1 OR #2 | 88,371 |
| #4 | “Harm Reduction"[Mesh] | 4,133 |
| #5 | "harm reduction"[Title/Abstract] OR "harm minimisation"[Title/Abstract] | 7,891 |
| #6 | #4 OR #5 | 9,547 |
| #7 | #3 AND #6 | 108 |

**Scopus (Elsevier) n=103**Date of search:

|  | **Query** | **Results** |
| --- | --- | --- |
| #1 | TITLE-ABS-KEY ( "self injur*" OR self-injur* OR "self harm" OR self-harm ) | 21,615 |
| #2 | TITLE-ABS-KEY ( "harm reduction" OR "harm minimisation" ) | 14,993 |
| #3 | #1 AND #2 | 103 |

**Sociological Abstracts (ProQuest) n=15**Date of search: August 10, 2023

|  | **Query** | **Results** |
| --- | --- | --- |
| #1 | MAINSUBJECT.EXACT("Self destructive behavior") | 2,703 |
| #2 | title(“self injury” OR self-injury OR “self harm” OR self-harm) OR abstract(“self injury” OR self-injury OR “self harm” OR self-harm) | 1,551 |
| #3 | #1 OR #2 | 3,147 |
| #4 | MAINSUBJECT.EXACT("Harm reduction") | 1,232 |
| #5 | title(”harm reduction” OR ”harm minimisation”) OR abstract(”harm reduction” OR ”harm minimisation”) | 1,825 |
| #6 | #4 OR #5 | 1,985 |
| #7 | #3 AND #6 | 15 |
